# Demographic Characteristics and Treatment Outcomes of Intracranial Atherosclerosis Stenting: A Retrospective Case-Series of 216 Consecutive Patients

**DOI:** 10.3390/jcm14010125

**Published:** 2024-12-28

**Authors:** Marat Sarshayev, Botagoz Turdaliyeva, Gulnur Tanbayeva, Shayakhmet Makhanbetkhan, Maxat Mussabekov, Dimash Davletov, Aiman Maidan, Mynzhylky Berdikhojayev

**Affiliations:** 1 “Joint-Stock Company” Central Clinical Hospital, Almaty 050060, Kazakhstan; sarshayev_m@snh.kz (M.S.); makhanbetkhan_sh@snh.kz (S.M.); mussabekov_m@snh.kz (M.M.); mynzhyl@gmail.com (M.B.); 2 Kazakhstan School of Public Health, Kazakhstan’s Medical University, Almaty 050060, Kazakhstan; turdaliyeva@kncdiz.kz; 3 Department of Public Health, Al-Farabi Kazakh National University, Almaty 050060, Kazakhstan; gulnur.tanbayeva@kaznu.edu.kz; 4 Faculty of Medicine, Asfendiyarov Kazakh National Medical University, Almaty 050060, Kazakhstan; davletov.d@kaznmu.kz; 5 National Centre for Neurosurgery, Astana 010000, Kazakhstan; 6 Hospital of the Medical Center of the Administration of the President of the Republic of Kazakhstan, Astana 010000, Kazakhstan

**Keywords:** intracranial atherosclerosis, Kazakh population, hypertension, diabetes mellitus, dyslipidemia, comorbid conditions, cerebrovascular disease, intracranial stenting, DAPT

## Abstract

**Background/Objectives:** Intracranial atherosclerosis (ICAS) is a major cause of ischemic stroke, disproportionately affecting populations with significant vascular risk factors. Although ICAS imposes a considerable health burden, research on this condition in Central Asia remains scarce, especially among the Kazakh population. This study analyzes demographic characteristics, treatment outcomes, and procedural challenges associated with ICAS in 216 patients treated at a single institution. **Methods:** This retrospective study included patients with ≥70% intracranial artery stenosis confirmed by imaging and presenting with ischemic symptoms. All patients underwent angioplasty and stenting with dual antiplatelet therapy (DAPT). Data collected included demographics, comorbidities, stenosis characteristics, procedural details, and outcomes assessed by the modified Rankin Scale (mRS). **Results:** The median age was 63.5 years (IQR: 57–68.6), and 73.7% were male. Hypertension was the most common comorbidity (98%), followed by ischemic heart disease (58%) and diabetes mellitus (40.9%). Multi-location ICAS was significantly associated with patients over 75 years of age (*p* = 0.025). Additionally, obesity and stenosis severity greater than 70% showed trends toward significance, with *p*-values of 0.064 and 0.079, respectively. Stenosis predominantly affected the internal carotid artery (54.5%) and vertebrobasilar system (31.6%). The average hospital stay was longer for posterior circulation stenosis (7.1 days) compared to anterior circulation (4.7 days). The periprocedural complication rate was 0.7%, with two deaths attributed to ischemic complications. At follow-up, four patients experienced worsening mRS scores (>2), particularly those with severe stenosis in the basilar artery and M1 segment. **Conclusions:** ICAS in the Kazakh population is strongly associated with hypertension and aging, with posterior circulation stenosis contributing disproportionately to worse outcomes. The low complication rates highlight the safety of modern endovascular techniques. However, further research is needed to optimize treatment strategies for severe and multi-location ICAS, particularly in Central Asian populations.

## 1. Introduction

Intracranial atherosclerotic stenosis (ICAS) is a significant contributor to the global burden of cerebrovascular disease, accounting for a substantial proportion of ischemic strokes worldwide. Stroke was presented as the third-leading cause of death worldwide with a 10.7% death cause among all fatal records in the latest Global Burden of Disease, Injuries, and Risk Factors Study (GBD 2021) [1]. Epidemiological data suggest that 20 to 40 individuals per 100,000 worldwide experience an ischemic event related to intracranial atherosclerotic disease (ICAD) [2]. Postmortem examination of intracranial arteries has revealed a high prevalence of severe ICAD, with rates reaching 43% among individuals aged 60 to 69 years, 65% in the 70 to 79 age group, and 80% in those 80 years and older. These findings underscore the increasing burden of ICAD with advancing age [3].

Atherosclerosis is a progressive disease characterized by the accumulation of lipid deposits within the arterial walls, leading to vessel stenosis and ischemia [4]. Although Kazakhstan is considered an upper-middle-income country, the burden of stroke is much higher in comparison with other upper-middle-income countries and in some occasions can be compared with lower-middle-income countries [1]. In Kazakhstan, where the prevalence of cardiovascular diseases is notably high, studying the regional burden of intracranial atherosclerosis and its association with comorbid conditions such as hypertension, diabetes, and dyslipidemia is crucial for improving healthcare outcomes [5]. Stenting is widely used as a management strategy for patients with ICAS worldwide and has been in use in our institution since 2016 [6]. Understanding this condition’s demographic characteristics and treatment approaches is essential for guiding clinical management and enhancing patient care [7,8]. This study aims to comprehensively analyze the demographic and clinical characteristics of 216 patients with intracranial atherosclerotic stenosis in the Kazakh population, treated at a single institution.

## 2. Materials and Methods

**Figure 1:**
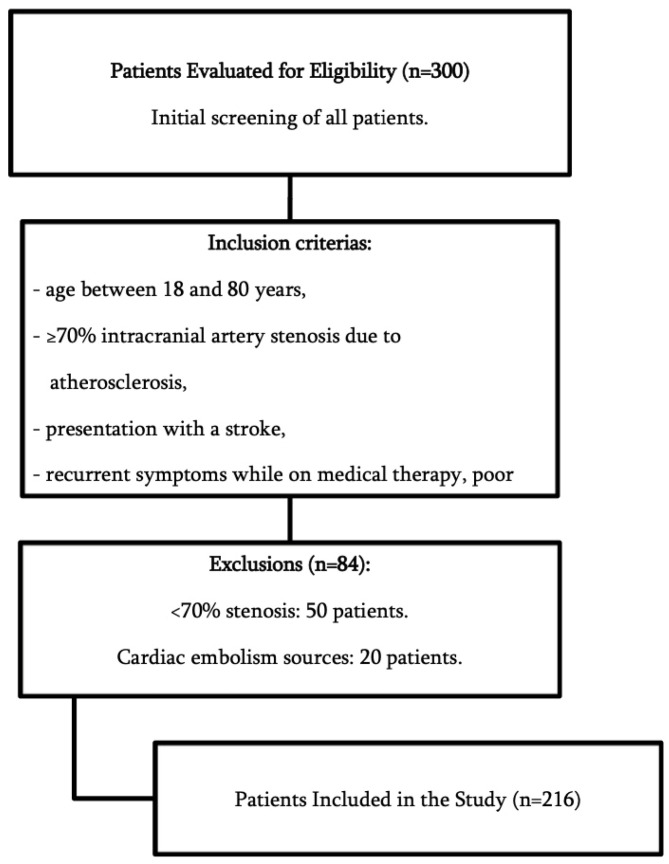
Inclusion and exclusion flowchart.

Study Design and Setting: This retrospective observational study included 216 patients treated in a single institution in Almaty, Kazakhstan. This institution has a national status, which explains the presence of patients from different regions of the country. These patients were enrolled at a single institution on a non-urgent basis from January 2016 to December 2023. Data were collected during the hospitalization period with a direct follow-up in the following 3, 6, and 12 months after the discharge. Participants: Patients were selected based on ≥70% intracranial artery stenosis confirmed through imaging (DSA, CTA, MRI/MRA) and presentation with ischemic symptoms. According to the TOAST (Trial of Org 10,172 in Acute Stroke Treatment) classification, these patients were defined as large-artery atherosclerosis subtypes. Patients were eligible for enrolment if they met the following criteria: age between 18 and 80 years, ≥70% intracranial artery stenosis due to atherosclerosis, presentation with a stroke, recurrent symptoms while on medical therapy, poor collateral flow according to CT perfusion or DSA, and more than 7 days after their stroke. The exclusion criteria encompassed non-compliance with prescribed medications, the presence of hemorrhagic infarction within a month before enrolment, and the identification of cardiac embolism sources. Figure 1 demonstrates the inclusion and exclusion flowchart. Table 1 summarizes the baseline clinical features of the participants. Variables: The study collected data on patient characteristics such as age, sex, and comorbidities (e.g., diabetes mellitus, hyperlipidemia, hypertension, ischemic heart disease). Additional information included the history of cerebral infarction or TIA, lifestyle factors like smoking, stenosis location and severity, treatment methods, stent type, residual stenosis, periprocedural complications, follow-up duration, in-stent restenosis, and pre-and post-treatment mRS scores. Data Sources and Measurements: All patients underwent balloon angioplasty followed by stent placement. Before stent placement, all participants underwent a regimen of dual antiplatelet therapy consisting of aspirin and clopidogrel for 3 to 5 days. Antiplatelet resistance testing was recommended, and in cases of detected resistance, the regimen was adjusted to include ticagrelor. Following stent implantation, dual antiplatelet therapy alongside statin treatment was maintained for six months. In the absence of antiplatelet resistance, a transition to monotherapy with clopidogrel for 12 months was advised.

### 2.1. Description of Operation

Femoral artery access with a 6F-8F sheath and guide catheter was obtained, and the flow was carefully inspected for collaterals. Patients received heparinization to an activated clotting time of 250 to 300 s, as well as pre-procedural dual antiplatelet therapy. After passing the lesion under roadmap guidance with a microcatheter, using a 300 cm 0.014-inch exchange wire, the balloon was advanced over the exchange wire while stabilizing the wire to prevent movement. A balloon size with a nominal diameter at 6 atmospheres was chosen, of approximately 80% of the true luminal diameter or 60% in lesions directly adjacent to angiographically visible perforators. The distal portion of the artery beyond the stenosis is typically 0.5–0.25 mm smaller in diameter compared to the normal segment, and the balloon used for angioplasty would be approximately 2 mm in diameter if the artery measures 2.5 mm. Under-dilation was advised to avoid arterial dissection, vessel rupture, and the “snowplow effect” of compressed plaque into perforator arteries. Careful monitoring of the distal exchange wire was recommended during stent delivery. The stent’s diameter was selected to match the indicated size on the packaging and inflated to a nominal pressure of 6 atmospheres. If a pressure of 14 atmospheres is specified, the diameter of the stent may be larger, facilitating subsequent post-dilation by approximately 0.25 less than the original stent diameter. Post-stenting balloon dilation within the stent was discouraged unless the residual stenosis remained ≥50% after stenting. Under road map guidance, a micro-guide wire was navigated through the stenotic segment, and a stent was sent to the stenosis for deployment. Patients were monitored in the Neuro Critical Care unit for 24 h after the procedure, with careful management of blood pressure to minimize the risk of reperfusion hemorrhage.

### 2.2. Stent Selection

Self-expanding stents are the preferred choice for the middle third-distal part of the basilar artery, as well as the A1, M1, M2, and P1 segments. A preferred option is a 5 mm diameter Credohill stent, which can be placed in an artery with a 1.5 mm diameter. A wall thickness of 2 mm is recommended, although it is worth considering that a smaller vessel diameter may result in a greater radial force. No complications were observed, and in most cases, the stent effectively eliminated the stenosis without the need for angioplasty. When coronary stents are used to treat stenosis in the internal carotid artery, proximal third of the basilar artery, or vertebral artery, the stent diameter should be selected to be approximately 0.25 mm less than the nominal diameter of the target artery.

### 2.3. Outcome Measures

Patients or their authorized proxies were contacted in person at 3, 6, and 12 months after discharge to collect data on functional status and quality of life. Any death was verified by examining the hospital medical records or local citizen registry. At discharge and 1 year after stroke onset, daily activities were assessed by mRS. Functional dependence was defined as mRS > 2. Table 2 defines the characteristics of outcomes.

### 2.4. Handling of Missing Data

In this study, missing data were evident for certain variables, including stenosis severity and postoperative complications. To ensure transparency and maintain the validity of the analyses, the following approach was adopted: Missing data points were identified during data collection and are summarized in Table 3, with the frequency and percentage of missingness for each variable explicitly reported. This allowed readers to understand the extent of missing data within the dataset. Given the relatively small proportion of missing data, no imputation methods were applied. This decision was made to avoid introducing bias or making assumptions about the missing values, which could potentially distort the findings. The effect of excluding cases with missing data was evaluated to ensure it did not significantly alter the overall trends or conclusions. The remaining sample sizes for each analysis were deemed sufficient to maintain statistical power.

### 2.5. Ethical Consideration

Written informed consent was obtained from the patient for publication and any accompanying images. A copy of the written consent is available for review by the Editor-in-Chief of this journal on request. The Ethical Committee of the JSC Clinical Hospital provided ethical approval for this study: Number 7 of ethical approval for Neurosurgery on 12 December 2023. In addition, the investigators ensured that the study conforms to the principles of the Declaration of Helsinki 78 (last revised in 2013) and that it was conducted per the ICH Guideline for Good Clinical Practice.

### 2.6. Statistical Methods

Statistical calculations were performed using SAS University Edition, version 3.8 (SAS Institute Inc., Cary, NC, USA). Statistical analysis included a chi-square test, and the observations were expressed as frequency and percent. *p* < 0.05 was considered statistically significant. Univariable and multivariable logistic regression analyses were performed to explore risk factors associated with mRS 3–6 outcome. The forest plots were made in Python 3.12 (Python Software Foundation, Beaverton, OR, USA). Continuous data were transformed to categorical by grouping through a cut-off point. For cholesterol, 5.6 mmol/L was chosen due to the reference ranges by the diagnostics laboratory. The age grouping was influenced by the European Society for Vascular Surgery (ESVC) guidelines as patients over 75 are risk-stratified as a group with more severe outcomes [9]. The mean number of hospitalization days in our hospital was 5 days; hence, this value was chosen as a cut-off value.

## 3. Results

The cohort had a median age of 63.5 (57–68.6) at the index stroke admission, and 73.7% were male. The mean age of patients gradually increased from 59 years in 2016 to 64 years in 2023, as seen in Table 1. The anatomical distribution of the patient cohort and their corresponding outcomes are detailed in Table 2. Of the 216 patients, 98% had a confirmed diagnosis of hypertension, 40.9% were diagnosed with diabetes mellitus, and 28% had dyslipidemia. The incidence of ischemic heart disease was found in 58% of the participants. Of all patients, 34% were smokers. The analysis showed a significant association in patients over 75 years old with intracranial stenosis in more than one location (*p* = 0.025), indicating an increased frequency in older patients. Moreover, patients with stenosis occlusion over 70% (*p* = 0.079) and obesity (*p* = 0.064) showed a trend toward significance. In contrast, sex, post-surgery complications, history of smoking, DM, IHD, arterial hypertension, and elevated cholesterol were not significant factors for multi-location ICAS (all *p* > 0.2), as seen in Table 3. Additionally, patients with multiple comorbidities exhibited more severe stenosis of the intracranial vessels, as measured by imaging techniques.

The target artery distribution was 54.5% internal carotid artery, 12.3% middle cerebral artery, 21.2% vertebral artery, 10.4% basilar artery, and 1.1% posterior cerebral artery. Patients with vertebrobasilar stenosis accrued a total of 615 hospital days, equating to an average length of stay of 7.1 days, compared to 4.7 days for those with stenosis in the anterior circulation. There was no significant association between stenosis quantity and hospitalization stay length (*p* = 0.214).

The study utilized a variety of stent types, including coronary stents: Ultimaster (Terumo Corporation, Tokyo, Japan) in 135 cases, Resolute Integrity, Onyx (Medtronic Inc., Santa Rosa, CA, USA) in 19 cases, Promus PREMIER (Boston Scientific Corporation, Marlborough, MA, USA) in 11 cases, and BioMime (Meril Life Sciences Pvt. Ltd., Vapi, Gujarat, India) in 1 case, Supraflex (Sahajanand Medical Technologies Ltd., Surat, India) in 1 case; and self-expandable stents: Acclino flex (Acandis GmbH, Pforzheim, Germany) in 67 cases, Neuroform Atlas (Stryker Corporation, Fremont, CA, USA) in 12 cases, Xience Xpedition (Abbott Vascular, Santa Clara, CA, USA) in 11 cases, Orsiro (BIOTRONIK SE & Co., Berlin, Germany) in 11 cases, Credo (Acandis GmbH, Pforzheim, Germany) in 8 cases, Solitaire (Medtronic Inc., Santa Rosa, CA, USA) in 2 casesand LEO (Balt Extrusion, Montmorency, France) in 1 case.

### 3.1. Clinical Outcomes

During the 72-h periprocedural period following the procedure, two deaths from strokes were reported, resulting in a 0.7% periprocedural complication rate. These fatalities occurred in patients with baseline stenosis of 90% or greater in the left vertebral artery. One death was attributed to ischemic complications, while the other resulted from intraventricular tamponade, classified as a hemorrhagic complication.

Our study reported various complications that occurred during or shortly after the procedures. Three patients experienced immediate thrombosis, necessitating the removal of the implanted stents. Among these, one individual with 75% basilar artery stenosis who underwent stenting exhibited a worsening in modified Rankin Scale scores from 3 to 4, while another patient with 95% left vertebral artery stenosis showed an mRS increase from 2 to 4 due to pontine ischemia. Additionally, a patient with 70% left vertebral artery stenosis experienced acute intestinal bleeding, resulting in a deterioration in mRS scores from 4 to 5.

An additional patient with 80–89% right cavernous stenosis who underwent stenting exhibited clinical deterioration, with an increase in modified Rankin Scale (mRS) scores from 3 to 5; this individual also presented with concurrent myocardial complications. Furthermore, another patient with 70% right vertebral artery stenosis who underwent stenting experienced an mRS score change from 3 to 4, which was attributed to non-adherence to prescribed medications.

Additional complications included a case of stent thrombosis that occurred on postoperative day 7 and a patient with right M2 stenosis who initially presented with aphasia, which resolved by postoperative day 3. Furthermore, a single instance of sigmoid and transverse sinus thrombosis was reported. Technical issues related to the stents were also documented, such as stent migration and the failure to deploy one stent due to vessel tortuosity.

The study highlighted a high proportion of mortality among patients with severe basilar artery stenosis. Notably, in three out of four patients who received coronary stents, clinical status worsened, and two out of three mortality cases were observed.

**Figure 2:**
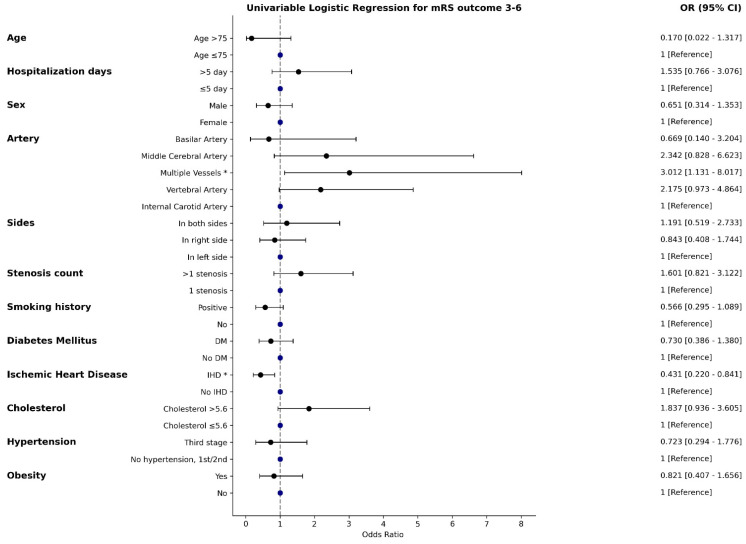
Univariable logistic regression results. * indicates statistically significant values (*p* < 0.05).

In the univariable analysis (Figure 2), stenosis in multiple vessels was significantly associated with increased odds (OR = 3.012, 95% CI: 1.131–8.017). Stenosis in the middle cerebral artery (OR = 2.342, 95% CI: 0.828–6.623) and vertebral artery (OR = 2.175, 95% CI: 0.973–4.864) showed trends toward higher odds but lacked statistical significance. Elevated cholesterol (>5.6 mmol/L) also indicated increased odds without significance. Conversely, ischemic heart disease demonstrated a protective effect, significantly reducing odds (OR = 0.431, 95% CI: 0.220–0.841), while smoking history and age >75 showed trends toward decreased odds but were not significant.

**Figure 3:**
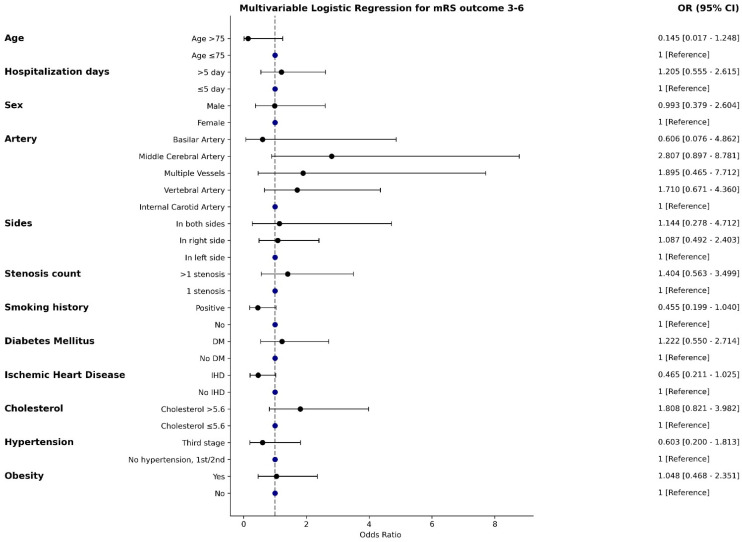
Multivariable logistic regression results.

In the multivariable analysis (Figure 3), the association of multiple vessel stenosis with increased odds was attenuated and lost significance, as well as the protective effect of ischemic heart disease. Other factors, including middle cerebral artery stenosis, stenosis severity, elevated cholesterol, age >75, and smoking history, remained consistent with univariable trends but without significance.

### 3.2. Case Examples

**Figure 4:**
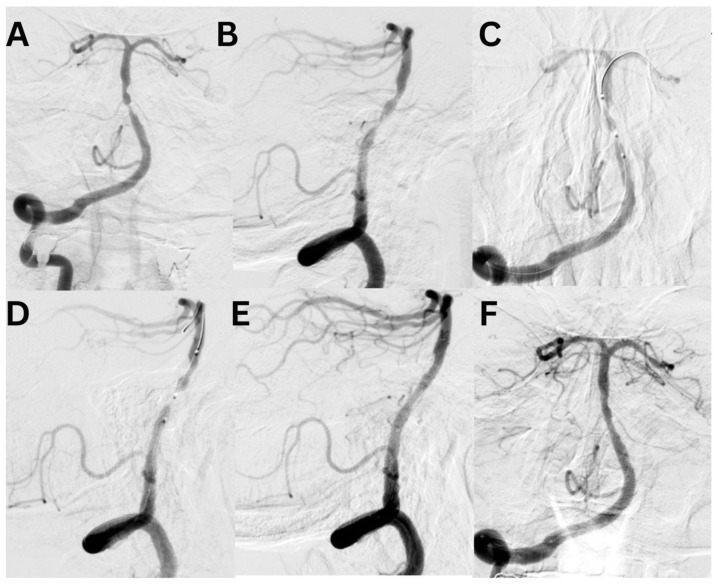
A diagnostic cerebral angiogram demonstrated an occlusion in the middle segment of the basilar artery on anteroposterior (**A**) and lateral (**B**) projections. Balloon angioplasty was performed, as shown in image (**C**), followed by deployment of a Credo stent, as seen in image (**D**). Subsequent control angiographies confirmed adequate stent placement and restoration of normal arterial flow on lateral (**E**) and anteroposterior (**F**) projections.

Case #1. A 75-year-old male patient with a history of myocardial infarction, type 2 diabetes mellitus, and hypertension presented with symptoms including slurred speech, temporary followed by full recovery, dizziness, tinnitus, unsteady gait, and lower extremity weakness. According to his wife, these symptoms began one week prior. Further diagnostic imaging with CTA revealed a subocclusion of the basilar artery, moderate stenosis in the V1 segment of the right vertebral artery, and hypoplasia of the left vertebral artery. Neurological examination at the time of admission was unremarkable. The patient was placed on dual antiplatelet therapy, and the VerifyNow test was performed, which detected no resistance. A surgical procedure was performed under local anesthesia. A 7Fr Fubuki guide catheter was inserted into the right vertebral artery, as seen in Figure 4. Under surgical guidance, an ASAHI Sion blue 0.014 × 180 cm microwire was advanced through the stenotic area. A NeuroSpeed 2 × 8 mm catheter balloon was then advanced along the microcatheter, and inflated twice for 2 s within the stenotic segment, resulting in a residual stenosis of up to 40%. Subsequently, a 5.0 × 25 mm Credo intravascular stent was deployed through the NeuroSpeed catheter balloon. Angiographic control confirmed the elimination of the stenosis, and the arteries and veins were found to be patent. At the 3-month follow-up, the patient reported no complaints, and MRI did not show any new signs of stroke.

**Figure 5:**
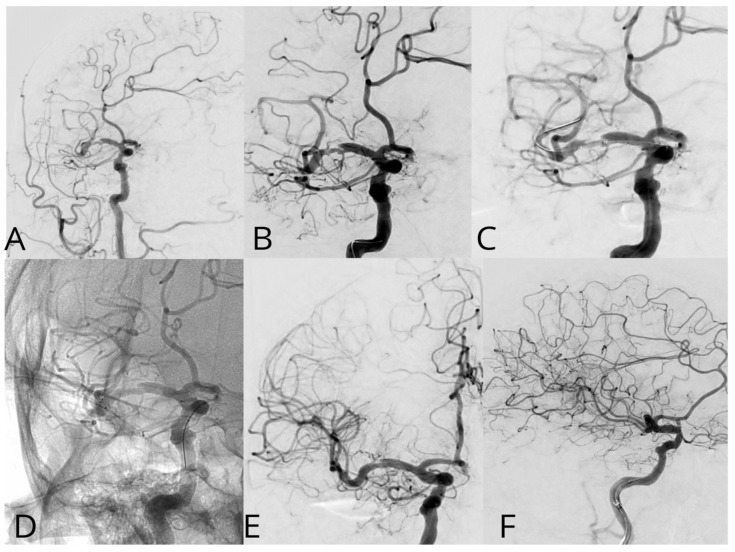
A diagnostic cerebral angiogram demonstrates an 80% stenosis of the right MCA (**A**,**B**) in a patient who presented with recurrent TIAs as evidenced by left-sided weakness/numbness. A diagnostic cerebral angiogram performed after stent placement and angioplasty demonstrates (**C**,**D**) resolution of stenosis. The operation was performed with an Acclino stent (**E**,**F**).

Case #2. A 48-year-old male patient presented with persistent pulsatile sensation in the parietal region, tinnitus, dizziness, blood pressure instability, right-sided weakness, general weakness, decreased work capacity, sleep disturbances, and memory loss. The patient had a history of hypertension for over 10 years and had experienced an ischemic stroke in the left middle cerebral artery territory two months prior, which was managed conservatively. Magnetic resonance imaging revealed signs of chronic ischemia in the right parietal and temporal lobes with cystic-gliotic changes, as well as single foci of hemosiderin deposition in the white matter of the cerebral hemispheres and basal ganglia, likely indicating microhemorrhage in the chronic stage. The patient underwent a comprehensive evaluation by a multidisciplinary team, including a cardiologist, neurologist, and pharmacologist, and was subsequently placed on ticagrelor. The procedure was performed under local anesthesia. An 8 French introducer was placed in the right femoral artery, and 8 French Hyperion catheters were navigated and positioned in the left internal carotid artery, covering the area of dissection and stenosis. DSA revealed a left M1 segment stenosis (Figure 5A,B). An Asahi Chikai Black 0.014 microwire was then advanced and placed in the M2 segment of the left middle cerebral artery. A dual-lumen coaxial microcatheter, the NeuroSpeed 2.0 × 8.0 mm, was navigated to the stenotic region and inflated twice using an inflator, resulting in a residual stenosis of up to 40%. Next, during the attempt to deploy an Acclino Flex 5.0 × 20 mm stent through a balloon catheter starting from the M1 bifurcation of the left middle cerebral artery, the proximal portion of the stent became fixed within the balloon catheter due to the radiopaque markers. As a result, the balloon microcatheter and the damaged stent were removed (Figure 5C). Subsequently, a GAMA 17 catheter was navigated through the Asahi Chikai Black 0.014 microwire, and an intravascular Acclino Flex 5.0 × 20 mm stent was reinserted and deployed to eliminate the stenosis (Figure 5D). The final angiographic evaluation demonstrated a patent left middle cerebral artery without any residual narrowing (Figure 5E,F).

## 4. Discussion

Intracranial atherosclerotic stenosis (ICAS) remains a significant cause of ischemic stroke, especially in patients with a high burden of vascular risk factors. The Kazakh population, with the unique epidemiological and clinical characteristics of this region, is under-reported in the general literature [5]. The incidence of cerebrovascular disease in Kazakhstan has increased notably, rising from 258.4 cases per 100,000 individuals in 2015 to 433.7 cases per 100,000 in 2020. Additionally, official data suggest an average inpatient mortality rate from stroke of 16.2% within the country, while the average time for patients to reach the hospital following an emergency call is approximately 40 min [10]. This study provides valuable insights into the demographic characteristics, anatomical distribution, and clinical outcomes of ICAS treatment in a single institutional cohort of 216 patients, highlighting the interplay between patient-specific factors and procedural outcomes. The median age of patients in our cohort was 63.5 years, with a gradual increase in mean age over the study period from 59 to 64 years. This aligns with previous studies demonstrating that ICAS is predominantly a disease of older adults. The predominance of males (73.7%) is also consistent with prior reports that ICAS exhibits a male bias, attributed to differences in vascular risk profiles and hormonal influences [11,12].

Hypertension (98%), diabetes mellitus (40.9%), and ischemic heart disease (58%) were highly prevalent in our cohort, emphasizing their established role as key risk factors for ICAS. These findings mirror those from the Northern Manhattan Stroke Study and other population-based studies, which consistently identify hypertension as the most prominent risk factor [13,14]. Furthermore, our data suggest an association between age over 75 years and multi-location ICAS, as well as a trend toward significance for obesity and stenosis severity, underscoring the contribution of metabolic syndrome to the progression of ICAS [15]. Obesity is well established as a key contributor to atherosclerosis through mechanisms such as chronic inflammation, insulin resistance, and endothelial dysfunction. Studies have shown that obesity increases the risk of both intracranial and extracranial atherosclerosis, potentially through systemic vascular injury and lipid metabolism dysregulation [12]. While our findings did not achieve statistical significance, they are consistent with evidence from large cohort studies indicating a higher burden of cerebrovascular disease in obese individuals.

The distribution of stenosis in our cohort showed the internal carotid artery (ICA) as the most frequently affected vessel (54.5%), followed by the vertebral artery (21.2%) and middle cerebral artery (12.3%). This predominance of anterior circulation stenosis is in line with autopsy studies and imaging analyses, which report ICA and MCA as the most involved vessels in ICAS [16]. However, patients with posterior circulation stenosis, particularly in the vertebrobasilar territory, exhibited a longer hospital stay (7.1 days on average), reflecting the more severe clinical presentations and poorer outcomes associated with posterior circulation strokes [16,17]. Severe stenosis is a marker of advanced atherosclerotic disease and has been associated with increased ischemic stroke risk. Prior studies, including the SAMMPRIS trial, have demonstrated that patients with high-grade stenosis are at higher risk for recurrent stroke and worse outcomes. Moreover, posterior circulation stenosis, often associated with severe narrowing, carries a particularly poor prognosis due to the critical blood flow requirements in this region [18].

Our analysis demonstrated a low periprocedural complication rate of 0.7%, with two ischemic stroke-related deaths within 72 h. While the rate of complications is comparable to other reports in the literature, such as the SAMMPRIS trial, which reported a 2.5% perioperative stroke or death rate, it is notable that our study also documented unique complications such as stent thrombosis, hemorrhage with ventricular tamponade, and stent migration [18,19].

Of particular interest is the finding that worsening outcomes, defined as an mRS score > 2, occurred in four patients. The basilar artery, vertebral artery, and M1 segment were the most affected sites in patients with severe stenosis, consistent with studies indicating that posterior circulation and high-grade stenosis carry a higher risk of adverse outcomes [20,21]. Additionally, the choice of stent did not show a significant association with procedural success or hospital length of stay, supporting findings from prior meta-analyses that suggest procedural technique and patient factors may outweigh stent design in determining outcomes [22].

Our findings align with and expand upon the existing literature by demonstrating that older age, multiple comorbidities, and posterior circulation involvement are associated with worse outcomes in ICAS [23]. Studies like the Chinese Intracranial Atherosclerosis Study (CICAS) have similarly emphasized the role of vascular risk factors and lesion characteristics in determining stroke risk and prognosis [24]. However, our results suggest that modern endovascular techniques, coupled with careful patient selection, can achieve favorable outcomes even in patients with severe stenosis.

Stents such as the Pegasus, HPS 4.5 mm, and Credohill 5 mm, which are not coated with cytostatic agents, have a higher rate of restenosis in follow-up angiograms. Coronary stents are preferred as they have demonstrated a lower incidence of restenosis on follow-up angiograms; however, some complications might happen.

The existing studies have been limited by small sample sizes, lack of randomization, and inadequate participant selection and blinding. Nevertheless, the natural history of intracranial atherosclerosis indicates that specific subgroups may experience more severe disease progression, and these patient populations may potentially benefit from percutaneous transluminal angioplasty and stenting for long-term reduction in ischemic events, as demonstrated in the WEAVE trial. Furthermore, with the involvement of experienced interventionalists and appropriate patient selection following the on-label usage guidelines, the use of the Wingspan stent for intracranial atherosclerotic disease has exhibited a low periprocedural complication rate and an excellent safety profile. Notably, this represents the largest on-label, multicenter, prospective trial of the Wingspan stent system to date, with the lowest reported complication rate [25]. The WOVEN study followed up on a group of 129 patients for 1 year after they underwent stenting in accordance with the current guidelines. This patient population was more homogeneous than those in previous studies, and the results showed a relatively low 8.5% rate of stroke and death within 1 year after the stenting procedure [26].

A recent meta-analysis of four studies including 352 patients who failed medical therapy revealed that patients who underwent stent rescue after failed MT experienced significantly improved rates of favorable clinical outcomes (OR 2.87, 95% CI 1.77–4.66, *p* < 0.001) and lower mortality (OR 0.39, 95% CI 0.16–0.93, *p* = 0.03) at 90 days, without any increased risk of symptomatic intracranial hemorrhage (OR 0.68, 95% CI 0.37–1.27, *p* = 0.23), compared to non-stented patients who failed medical therapy. These findings suggest that in carefully selected patients with symptomatic intracranial stenosis refractory to medical management, endovascular stenting may be a viable option to improve clinical outcomes [27].

Ischemic heart disease and intracranial atherosclerosis frequently co-occur due to shared risk factors, such as hypertension, hyperlipidemia, diabetes mellitus, and smoking. Studies estimate that up to 20–30% of patients with ICAS also have concurrent coronary artery disease, which is characteristic of IHD [28].

The most common causes of death in patients with coexisting IHD and ICAS are acute myocardial infarction and large ischemic strokes. Studies suggest that the risk of fatal strokes is higher in patients with ICAS than in those with extracranial carotid stenosis. Moreover, heart failure due to progressive IHD also contributes significantly to mortality in this population [29].

In our analysis, a multi-vessel involvement was significantly associated with higher odds of the event compared to isolated internal carotid artery involvement. Additionally, our analysis reveals that a history of ischemic heart disease was significantly associated with lower odds of mortality compared to those without ischemic heart disease, a finding consistent in both univariable and multivariable analyses. These results emphasize the importance of considering multi-vessel involvement as a critical risk factor and highlight the protective association of ischemic heart disease in this context.

To our knowledge, this is the first presentation of ICAS demographics and outcome results in Kazakhstan, and Central Asia. The status of a national hospital, with patients across the country, allows us to generalize this study to a national-scale level; however, multicenter collaboration is necessary, especially in the Central Asian region, which is usually a white spot in the medical scientific world.

### Study Limitations

This study has certain limitations. The retrospective nature of the data collection carries the risk of incomplete or inconsistent recording, leading to information bias. Missing data on key variables, such as stenosis severity and postoperative complications, may have impacted the robustness of the statistical analyses. Additionally, the retrospective approach restricts the ability to control for confounding variables, affecting the interpretation of causal relationships. The relatively small sample size for certain anatomical locations or patient subgroups reduces statistical power and may result in imprecise estimates or failure to detect significant associations. This limitation could have hindered the identification of clinically relevant patterns or subgroup-specific outcomes. The absence of a control group prevents direct comparison with alternative treatment modalities or natural disease progression. Without a comparator, the efficacy and safety of the interventions cannot be conclusively determined relative to medical therapy or other interventions. Nevertheless, the analysis of data from 216 patients presenting with ICAS provides valuable insights, although the conclusions are not definitive. To improve future research, the following strategies could be considered: (1) Expanding the study to multiple healthcare institutions would capture a more diverse patient population, enhancing the generalizability of the findings. This could help identify regional or organizational differences in demographics, treatment practices, and outcomes, providing a more comprehensive understanding of intracranial atherosclerosis. (2) Adopting a prospective study design would enable standardized data collection and real-time monitoring, reducing the risk of information bias inherent in retrospective studies. This approach would also facilitate the collection of more detailed data on patient characteristics, procedural details, and long-term outcomes, improving the overall quality of evidence. (3) Incorporating control groups, such as patients treated with medical therapy alone, would enable direct comparisons of treatment efficacy and safety. This would provide stronger evidence for the advantages of stenting procedures relative to other management options. (4) Recruiting larger patient cohorts, particularly for underrepresented subgroups like posterior circulation stenosis, would increase statistical power and precision. This would help detect meaningful associations and identify subgroup-specific predictors of outcomes. (5) Integrating advanced imaging techniques and biomarkers could enhance the understanding of disease progression and treatment effects, potentially aiding in better patient selection and stratification. (6) Extending the follow-up period in future studies would provide insights into the durability of stenting outcomes, including rates of restenosis, long-term functional recovery, and quality of life. (7) Establishing collaborative research networks across institutions and countries would facilitate larger-scale, standardized data collection and analysis, helping to overcome limitations in resources and sample size at individual centers.

Future multicenter, prospective studies with larger cohorts and control groups are needed to validate these findings and explore the long-term outcomes of ICAS management. Such research will help refine treatment strategies and improve the care of patients with ICAS in diverse populations. Until then, the results should be interpreted as preliminary and hypothesis-generating, providing a foundation for further investigation.

## 5. Conclusions

The findings of this study provide valuable insights into the demographic characteristics and treatment outcomes of intracranial atherosclerotic stenosis (ICAS) in the Kazakh population. The results underscore the significant association of ICAS with hypertension and aging, as well as the disproportionate impact of posterior circulation stenosis on clinical outcomes. The low complication rates observed highlight the potential safety and efficacy of modern endovascular techniques for ICAS treatment.

## Figures and Tables

**Table 1:** Demographic characteristics of patients.

Mean Age	63.5
Area	
East Kazakhstan	6
West Kazakhstan	66
North Kazakhstan	10
South Kazakhstan	50
Almaty	84
Arterial Hypertension (present in)	213
Type 2 Diabetes (present in)	117
Dyslipidemia (present in)	85
Smoking (present in)	91
Ischaemic heart disease (present in)	152

**Table 2:** The anatomical distribution of the patient cohort, with stenosis severity and their corresponding outcomes.

Artery	Localisation	#	Degree Before Stenting	Outcome, on Discharge and at Latest FU
ICA	Right Petrous	7	70–79 (2)	mRS 1-1 mRS 4-4
			80–89 (5)	mRS 1-1 (4) mRS 2-2
	Left Petrous	7	70–79 (6)	mRS 1-1 (3) mRS 2-2 (3)
			90–99	mRS 1-1
	Right Cavernous	32	70–79 (20)	mRS 1-1 (8) mRS 2-2 (9) mRS 3-3 (3)
			80–89 (11)	mRS 1-1 (5) mRS 2-2 (3) mRS 3-4 mRS 4-4
			90–99	mRS 1-1
	Left Cavernous	35	70–79 (21)	mRS 1-1 (9) mRS 2-2 (5) mRS 3-3 (1) mRS 4-4 (6)
			80–89 (7)	mRS 1-1 (4) mRS 2-2 (2) mRS 3-3
			90–99 (7)	mRS 1-1 (2) mRS 2-2 (2) mRS 3-3 (3)
	Right Supraclinoid	16	70–79 (8)	mRS 1-1 (4) mRS 2-2 (2) mRS 3-3 mRS 4-4
			80–89 (5)	mRS 1-1 (2) mRS 2-2 mRS 3-3 (2)
			90–99 (3)	mRS 1-1 mRS 2-2 mRS 4-2
	Left Supraclinoid	30	70–79 (10)	mRS 1-1 (5) mRS 2-2 (4) mRS 4-4
			80–89 (13)	mRS 1-1 (9) mRS 2-2 (2) mRS 3-3 (2)
			90–99 (7)	mRS 1-1 (2) mRS 2-2 (3) mRS 3-3 mRS 4-4
	Left Ophtalmic	4	70–79 (2)	mRS 0-1 mRS 1-1
			80–89	mRS 1-1
			90–99	mRS 2-2
	Left Choroidal	1	90–99	mRS 2-2
Basilar artery		27	70–79 (9)	mRS 1-1 (4) mRS 2-2 (4) mRS 3-3 mRS 3-4
			80–89 (6)	mRS 1-1 (3) mRS 2-2 (3)
			90–99 (12)	mRS 1-1 (3) mRS 2-2 (3) mRS 2-3 (3) mRS 3-3 (3)
Vertebral artery	Left VA	32	70–79 (9)	mRS 1- 1 (3) mRS 2-2 (5) mRS 2-4
			80–89 (11)	mRS 1-1 (3) mRS 2-2 (3) mRS 3-3 (4) mRS 4-4
			90–99 (12)	mRS 1-1 (2) mRS 2-2 (5) mRS 3-3 (4) mRS 4-5 mRS 3-6 (2)
	Right VA	23	70–79 (5)	mRS 1-1 mRS 2-2 mRS 3-3 (2) mRS 3-4
			80–89 (11)	mRS 1-1 (5) mRS 2-2 (3) mRS 3-3 (2) mRS 4-4
			90–99 (7)	mRS 1-1 (5) mRS 2-2 mRS 4-4
Others	M1	29	70–79 (5)	mRS 1-1 (2) mRS 2-2 mRS 2-3 mRS 3-3
			80–89 (16)	mRS 1-1 (2) mRS 1-2 (2) mRS 2-2 (9) mRS 3-3 mRS 4-3 mRS 4-2
			90–99 (8)	mRS 1-1 (2) mRS 2-2 (2) mRS 3-3 (2) mRS 4-3 mRS 5-5
	M2	3	70–79	mRS 1-1
			80–89	mRS 2-2
			90–99	mRS 1-1
	P1	3	70–79	mRS 1-1
			90–99 (2)	mRS 2-2 (2)

**Table 3:** Analysis of factors associated with multi-vessel and single-vessel intracranial stenosis predisposition. Missing data frequency in stenosis severity (2), postoperative complications (36), diabetes mellitus (1); χ^2^—chi-square.

	Single Location Stenosis		Multiple Location Stenosis		Total	χ^2^	*p*-Value
	n	%	n	%	n		
Age							
≤75 years	147	73.87	52	26.13	199	5.5553	0.0184
Over 75 years	8	47.06	9	52.94	17		
Hospitalization length of stay							
≤5 days	57	77.03	17	22.97	74	1.5413	0.2144
>5 days	98	69.01	44	30.99	142		
Sex							
Female	29	64.44	16	35.56	45	1.5008	0.2205
Male	126	73.68	45	26.32	171		
Smoking history							
Absent	73	70.19	70	71.43	143	0.0373	0.8469
Present	31	29.81	28	28.57	59		
Postoperative complications							
Absent	123	73.21	45	26.79	168	1.2363	0.2662
Present	7	58.33	5	41.67	12		
Diabetes Mellitus							
Absent	80	73.39	29	26.61	109	0.1861	0.6662
Present	75	70.75	31	29.25	106		
Ischemic heart disease							
Absent	43	70.49	18	29.51	61	0.0674	0.7952
Present	112	72.26	43	27.74	155		
Hypercholesterolemia							
Absent	109	69.43	48	30.57	157	1.5431	0.2142
Present	46	77.97	13	22.03	59		
Arterial hypertension							
No AH, first or secon stage	18	62.07	11	37.93	29	1.5521	0.2128
3rd stage	137	73.26	50	26.74	187		
Obesity							
Absent	102	68	48	32	150	3.4234	0.0643
Present	53	80.3	13	19.7	66		

## Data Availability

The data presented in this study are available on request from the corresponding author.
